# Role of Non‐Thermal Processing Technologies in Modulating Protein Allergenicity in Legumes

**DOI:** 10.1002/fsn3.72369

**Published:** 2026-09-14

**Authors:** Viateur Ngabonzima, Flora G. C. Ekezie, Alberta N. A. Aryee, John O. Onuh

**Affiliations:** ^1^ Department of Food and Nutritional Sciences Tuskegee University Tuskegee Alabama USA; ^2^ SGS North America Bloomfield New Jersey USA; ^3^ Food Science & Biotechnology Program, Department of Human Ecology, College of Agriculture, Science and Technology Delaware State University Dover Delaware USA

**Keywords:** cold plasma, high‐pressure processing, IgE‐mediated allergy, non‐thermal processing, protein allergenicity

## Abstract

The global shift toward plant‐based diets has increased legume consumption due to health and environmental benefits. However, storage proteins in legumes, such as albumins, vicilins, and legumins, can cause immunoglobulin E (IgE)‐mediated allergic reactions ranging from mild symptoms to severe anaphylaxis. The objective of this review is to evaluate the role of non‐thermal processing technologies (NTTs) in reducing legume allergenicity. Current evidence indicates that NTTs such as pulsed electric fields, high‐pressure processing, ultrasound, cold plasma, irradiation, and enzymatic hydrolysis can induce structural modifications in allergenic proteins, leading to reduced IgE binding. Synergistic applications, particularly the combination of two or more NTTs, especially with enzymatic hydrolysis, alter protein structures and lower allergenicity even more effectively than stand‐alone NTT treatments. This approach provides an alternative solution to conventional heat treatments such as roasting, boiling, etc. by modifying protein structures while better preserving nutritional and sensory attributes. The primary allergenicity reduction mechanisms of NTTs include protein unfolding, aggregation, fragmentation, and epitope masking or destruction, with protein unfolding the most documented by numerous researchers. Nonetheless, effectiveness still varies by legume type, protein composition, and processing conditions. Future research would prioritize standardizing processing parameters, physiologically relevant digestion tests, and clinical validation in order to facilitate the production of safe and hypoallergenic legume‐based food products.

## Graphical abstract

Non‐thermal processing technologies (NTTs) reduce legumes' allergenicity while preserving quality and nutrients retention (PEF, pulsed electric field; CP, cold plasma; HPP, high pressure processing; US, ultrasound; IR, infrared).

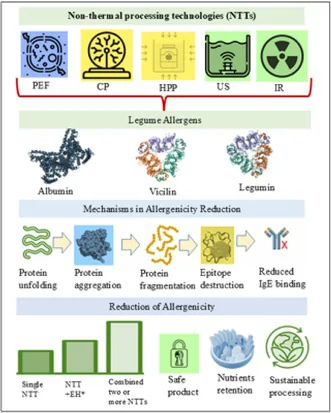

**Abbreviations**
ACP: Atmospheric Cold Plasma
CP: Cold Plasma
ELISA: Enzyme‐Linked Immunosorbent Assay
HPP: High‐Pressure Processing
IgE: Immunoglobulin E
ImmunoCAP: Immuno Solid‐Phase Allergen Chip
IUIS: International Union of Immunological Societies
LTP: Lipid Transfer Protein
NTTs: Non‐Thermal Technologies
OAS: Oral Allergy Syndrome
OVM: Ovomucoid
PEF: Pulsed Electric Field
SDS‐PAGE: Sodium Dodecyl Sulfate‐Polyacrylamide Gel Electrophoresis
SPT: Skin Prick Test
US: Ultrasound
WHO: World Health Organization

## 1 Introduction

The increasing global awareness of the benefits associated with plant‐based diets has contributed to a significant rise in the demand, including legumes, due to their recognized health advantages and positive environmental impact (Alcorta et al. 2021). A key consideration is whether such diets provide adequate nutrition and do not pose any health risk to humans. Legumes such as peanuts, peas, lentils, and soybeans are known for their high protein content, essential nutrients, and minerals (Becerra‐Tomás et al. 2019). Additionally, Kadyan et al. (2022) highlighted the role of legumes in supporting gut health, which can be attributed to their substantial fiber content.

While legumes are widely recognized for their nutritional and health benefits, they also present significant health risks due to their allergen content. Pumphrey and Gowland (2023) underscores health concerns, particularly for children, whose immune systems may react adversely to allergens found in various legumes such as peanuts, soybeans, chickpeas, and lupins. The allergic reactions triggered by these legumes can range from mild oral allergy syndrome (OAS) to life‐threatening anaphylactic reactions (Skypala 2019; Präger et al. 2023). Mastrorilli et al. (2024) highlighted in their study that seed storage proteins specifically vicilins (7S globulins), legumins (11S globulins), and albumins are the principal contributors to legume allergenicity. These proteins are notable for their resistance to both heat treatments and digestive enzymes, allowing them to persist through food processing and digestion. The presence of Immunoglobulin E (IgE)‐binding epitopes within these proteins is responsible for eliciting immune responses in sensitized individuals (Liu et al. 2024). Cross‐reactivity among legume species is another challenge, where people allergic to one type of legume may also experience allergenic reactions to other legumes because many legume allergens share structurally similar proteins with conserved IgE‐binding epitopes (Chentouh et al. 2022; Verma et al. 2013). For example, a person sensitized to peanut storage proteins such as Ara h 1 (a vicilin) may also react to lentil or pea proteins belonging to the same vicilin or legumin families due to similarities in their amino acid sequences and three‐dimensional structures.

Food processing methods can influence allergenicity of legumes (Gonzalez et al. 2025). However, conventional thermal approaches, including but not limited to boiling and roasting, often fail to eliminate allergenicity and may even enhance it by exposing or modifying epitopes (Verhoeckx et al. 2015). Thermal processing methods such as boiling, roasting, autoclaving, and steaming are largely applied for their contribution in enhancing the digestibility, safety, and sensory attributes of legumes (King et al. 2024). Pi et al. (2024) found that conventional thermal processing is often ineffective and inconsistent in lowering legume allergenicity. Additionally, the authors revealed a major limitation that heat‐stable allergenic proteins including vicilins, legumins, and albumins are able to retain their allergenic potential even after high‐intensity thermal treatment. In some cases, heat processing may expose hidden (cryptic) epitopes or induce Maillard reactions (protein‐sugar interactions), potentially increasing allergenicity by creating neo‐allergens (Ramchandran et al. 2016).

Numerous investigations have been undertaken to elucidate the allergenic properties of legumes and the influence of various processing techniques on the attenuation of such allergenicity (Verma et al. 2013). Nevertheless, these investigations have been limited by an excessive reliance on thermal processing methodologies, a pronounced dependence on in vitro IgE‐binding assays, disjointed species‐specific studies, insufficient mechanistic understanding, and variable parameters pertaining to non‐thermal processing, a lack of comprehensive evaluation of synergistic technologies, and a dearth of clinical validation. Previous reviews have focused primarily on thermal processing, lacked mechanistic comparisons across NTTs, or did not address synergistic approaches. This review provides the first cross‐legume mechanistic framework correlating structural protein alterations with immunological outcomes. Although many studies have explored individual non‐thermal technologies, the present evidence remains fragmented since the reductions in allergenicity are often reported using different biomarkers, processing conditions and parameters, and legume species. Therefore, this makes direct comparisons difficult among technologies, and it remains unclear whether observed reductions principally arise from protein unfolding, aggregation, oxidation, or the synergistic effect of numerous mechanisms. The authors recommend that non‐thermal technologies be interpreted according to their respective major structural effects on allergenic proteins instead of viewing them as isolated interventions. As a result, a common framework for comparing technologies and identifying processing strategies with greatest translational potential for hypoallergenic legume foods.

In a systematic review carried out by Arteaga‐Marin et al. (2025), it was found that the application of non‐thermal technologies (NTTs) presents promising alternatives for reducing allergens compared to traditional thermal methods. These NTTs include pulsed electric field (PEF), high‐pressure processing (HPP), cold plasma (CP), ultrasound (US), and enzymatic hydrolysis, among others (Arteaga‐Marin et al. 2025). Unlike heat‐based treatments, NTTs aim to modulate the structural integrity of allergenic proteins without significantly compromising nutritional, functional, and/or sensory qualities. The objective of this review, therefore, is to assess the effectiveness of NTTs at lowering or modifying the protein allergenicity in legumes. Such advancements could decrease allergic reactions among sensitive individuals while improving utilization of legumes' proteins which are increasingly important in plant‐based diets.

## 2 Methodology

The present review synthesizes available evidence on the application of non‐thermal technologies (NTTs) for the reduction of legume protein allergenicity. Relevant peer‐reviewed studies were identified through comprehensive literature searches of major scientific databases, including PubMed, Scopus, Web of Science, and Google Scholar, using combinations of keywords, such as legume allergy, protein allergenicity, non‐thermal processing, high‐pressure processing, pulsed electric field, cold plasma, ultrasound, IgE binding, and food allergens. Original research articles, review papers, and official reports published principally in English were given priority. The selected literature was critically evaluated and organized according to the type of non‐thermal technology, underlying mechanisms of allergenicity reduction, structural modifications of allergenic proteins, immunological outcomes, and reported effectiveness across different legume species. Particular attention was given to studies describing changes in IgE‐binding capacity, protein conformation, and epitope modification, while highlighting current knowledge gaps and future research priorities.

## 3 Protein Allergenicity in Legumes

Legume allergenicity is primarily attributed to seed storage proteins, including vicilins (7S globulins), legumins (11S globulins), and albumins, which are highly stable against heat and digestion (Bar‐El Dadon et al. 2013). These proteins can elicit IgE‐mediated immune responses in sensitized individuals and are often cross‐reactive among different legume species due to their structural similarities. This presents considerable challenges in managing legume allergies, particularly within food processing and dietary planning contexts (Chentouh et al. 2022). Table 1 describes the various sources and types of allergies as well as their symptoms and case studies.

**TABLE 1:** Sources and types of allergies as well as their symptoms and case studies.

No	Source of allergies	Types of allergies	Symptoms	Case studies	References
1	Air pollution, atopy, pollen exposure, house dust mites, mold spores, animal/pest dander	IgE‐mediated mechanisms: seasonal allergic conjunctivitis (SAC), and perennial allergic conjunctivitis (PAC); Non‐IgE‐induced mechanisms: blepharoconjunctivitis (CBC), VKC, and AKC.	Itching, redness and swelling, exacerbations	Allergic conjunctivitis: review of current types, treatments, and trends	Tariq (2024)
2	Foods (tree nuts, milk, eggs, shellfish, peanuts, wheat, soy)	Type I IgE‐mediated food allergy; some non‐IgE or mixed.	Skin hives, angioedema, vomiting, diarrhea, abdominal pain, throat tightness, hypertension, and anaphylaxis	Living with and caring for people with multiple food allergies: A Qualitative study	Ciaccio et al. (2024)
3	Medicinal drugs	Drug allergy	Respiratory distress, urticaria, angioedema, anaphylaxis, headache, chest and pelvic pain, acute fever	Drug Allergy	Jeimy et al. (2024)
4	Contact allergy	Allergic contact dermatitis, photo contact dermatitis, Contact urticaria, irritant contact dermatitis	Pain, burning, itching, anaphylaxis	Pediatric Contact Dermatitis	Silverberg (2025)

### 3.1 Major Allergens in Legumes

Legumes constitute a vital source of plant‐based protein, dietary fiber, and a broad array of micronutrients worldwide (Messina 2014). Despite their nutritional benefits, a good number are recognized to elicit allergenic reactions, with a pronounced impact on pediatric populations (Verma et al. 2013). Peanuts ( *Arachis hypogaea* ) and soybeans ( *Glycine max* ) are among the most common culprits, often eliciting severe IgE‐mediated reactions including mild to severe anaphylaxis. Additional allergenic sources including pulses such as lentils, chickpeas, peas, and lupins are commonly consumed across Mediterranean and South Asian regions (Verma et al. 2013; Costa et al. 2020). These legumes contain structurally related storage proteins, notably vicilins, legumins, and albumins, substantially contributing to their allergenic potential and cross‐reactivity. Understanding the specific allergenic proteins in major legumes is essential before evaluating processing interventions.

#### 3.1.1 Peanuts

Peanuts ( *Arachis hypogaea* ) offer numerous benefits worldwide, including their nutritional value as a source of protein, healthy fats, and essential vitamins (Arya et al. 2016). Additionally, peanuts are used in a variety of products, from food items to industrial applications including oil, protein meal, peanut butter, and snack foods, underscoring their versatility and economic significance (Arya et al. 2016; Machado et al. 2025). However, peanut allergens adversely affect the health and quality of life of millions of individuals globally. The seeds of the peanut plant contain an array of allergens that are able to induce the production of specific IgE antibodies in pre‐disposed individuals (Palladino and Breiteneder 2018). Only 16 peanut allergens are recognized by the World Health Organization/International Union of Immunological Societies (WHO/IUIS) Allergen Nomenclature subcommittee and these include cupins (Ara h 1 & Ara h 3), 2S albumins (Ara h 2, Ara h 6 & Ara h 7), Profilin (Ara h 5), PR‐10 (Ara h 8), non‐specific lipid transfer protein (LTP) Type 1 and Type 2 (Ara h 9, Ara h 16, and Ara h 17), oleosins (Ara h 10, Ara h 11, Ara h 14, and Ara h 15), and defensins (Ara h 12, and Ara h 13) (Palladino and Breiteneder 2018). Of all the peanut allergens, Ara h 1, Ara h 2, Ara h 3, and Ara h 6 were reported to be the mostly clinically relevant allergens (WHO/IUIS 2025). Peanut allergy symptoms can vary from mild to severe, including life‐threatening anaphylactic reactions (Novembre et al. 2024; Al‐Muhsen et al. 2003). Approximately 2% of the United States population are affected by peanut‐associated allergies (Warren et al. 2021). Although fatal outcomes are rare, peanut allergy remains a major public health concern because it is one of the leading causes of food‐induced anaphylaxis and can result in life‐threatening reactions (Ma et al. 2014). Researchers have extensively identified peanut allergens over the last decade (Schwager et al. 2015; Petersen et al. 2015; Mattsson et al. 2021).

#### 3.1.2 Soybean

Soybean ( *Glycine max* ), a member of the Fabaceae family, is recognized as a significant source of protein and phytochemicals, including isoflavones and phenolic compounds (Messina 2014). Its peptides demonstrate diverse biological functions, such as anti‐inflammatory, anticancer, and antidiabetic activities (Zhu et al. 2023). However, soybean is also considered a major allergenic food (Wiederstein et al. 2023). Among the main identified soybean allergens (Gly m4 and Gly m Bd 30K), Gly m 4 has been shown to elicit allergic reactions, typically manifesting as localized effects on the skin, gastrointestinal tract, or respiratory system (Finkina et al. 2022). Soybean allergens are classified as complete food allergens, capable of inducing specific IgE production and causing reactions ranging from mild rashes to severe anaphylaxis (Julka et al. 2012).

Soybean allergy is only predominant in certain geographic areas: 2% in Japan (Akiyama et al. 2011), 0.6% in the US (Gupta et al. 2019; Hultquist et al. 2022), and 0.5% in Europe (Spolidoro et al. 2023) according to self‐reported surveys. However, self‐reported surveys may not reflect the true prevalence of soybean allergy, as they can overestimate it by including unconfirmed adverse food reactions or intolerances and underestimate it due to recall bias, lack of symptom recognition, or failure to report mild or resolved allergies. Eight official soybean allergens are listed in the WHO/IUIS Allergen Nomenclature Sub‐Committee database (Wiederstein et al. 2023). Eight additional IgE‐binding proteins from soybean are also listed in the Allergome database. Anaphylactic reactions toward soy are severe for around one third of the cases and are more frequent in adults when compared to children (Dölle‐Bierke et al. 2023). They are mostly associated with Gly m 3 to 6 (Holzhauser 2022; Ito et al. 2011), and some case studies even suggest exercise as a co‐factor of anaphylaxis to soybean allergens, for instance, food‐dependent exercise‐induced anaphylaxis (FDEIA) (Hayashi et al. 2020; Adachi et al. 2009). Although sensitization toward profilin (Gly m 3) and PR10 protein (Gly m 4) was the most frequent cause for systemic reactions in a large retrospective study (Asero et al. 2021), Gly m 4 seems to be the predominating trigger (Mittag et al. 2004; Kleine‐Tebbe et al. 2002). While it is well‐known that the storage proteins Gly m 5 to 8 are resistant to heat and/or digestion (Riascos et al. 2016; Lin et al. 2006), Gly m 4 was only recently shown to refold after heating and trigger an IgE response, with or without homologous Bet v 1‐related epitopes (Finkina et al. 2022). Consequently, this justifies the preference for NTTs use as they may prevent Gly m 4 from refolding and hence limit its health‐related adverse effects. Furthermore, this allergen can cross the intestinal barrier, especially if the gastric pH is too high for proper digestion (Finkina et al. 2022). This might explain why soybean‐based drinks can be dangerous for people with birch or alder pollen allergy (Evrard et al. 2022).

#### 3.1.3 Beans

Red kidney bean ( *Phaseolus vulgaris* L.) is a protein rich legume that is consumed worldwide (Yan et al. 2026). Phaseolin is the most abundant allergen of kidney bean (Kumar et al. 2014). Allergy toward beans is rare and is frequently accompanied by IgE cross‐reactivity to other legumes. Although beans have fewer known allergens, cross‐reactivities with soybeans and peanuts have been reported (Cabanillas et al. 2018). The route of sensitization includes the oral mucosa and the respiratory tract as well as the skin (contact urticaria), although only one case has been reported for the latter: An 84‐year‐old man experienced skin swelling after skin contact with runner bean plants ( *Phaseolus coccineus* ) in his garden (Chentouh et al. 2022). Bean allergy highly depends on the genus and species of the bean. For instance, allergic reactions to the white bean ( *Phaseolus vulgaris* ) were highlighted by recent case reports. Matsui et al. (2023) reported an allergic reaction to white beans in a child. IgE‐reactivity determined by ImmunoCAP (a common in vitro diagnostic test used to measure specific IgE antibodies in a patient's blood to identify sensitization to particular allergens) occurred to bean allergens such as phyto‐hemagglutinin, Group 3 Late Embryogenesis Abundant proteins (LEA), lipoxygenase, and legumin. Another report published in 2023 described two children exhibiting allergic reactions to both white and red beans, yet showed no reactions to other legumes. Western blot analysis using sera of two patients that reacted to extracts from white and red beans identified two IgE‐binding proteins in the extracts with molecular weights in the ranges of 47–50 kDa and 28–31 kDa (Laiseca García et al. 2023).

Mung bean ( *Vigna radiata* ) is used in meat‐substitution products, which are consumed worldwide (Tarahi 2024). Two cases of OAS have been described, one in a teenage Japanese girl and another involving a male in his twenties (Kobayashi et al. 2023). Both cases tested positive for IgE specific to soybean Gly m 4 and birch Bet v 1 (Mittag et al. 2005). Lima beans ( *Phaseolus lunatus* ) induce positive skin test reactions in patients with other legume allergies but with a low percentage (3.4%) as shown by a survey for asthma and rhinitis patients in Delhi (Dhyani et al. 2007), which mostly correlates to cross‐reactivity with other legumes (Bernhisel‐Broadbent and Sampson 1989). However, case reports represent rare events and population‐level risk remains low.

#### 3.1.4 Peas

The high protein content in peas ( *Pisum sativum* ) makes them an important nutritional source (Wu et al. 2023). Their ability to absorb water has led to their widespread use in the production of meat‐substitution products (Shanthakumar et al. 2022). Due to the rising use of peas as an alternative to soy and wheat in plant‐based meats (Maningat et al. 2022; Zhang et al. 2024), case reports for pea allergy have been more frequent recently, including incidences of severe reactions, which indicate that a switch to a plant‐based diet can harbor risks for certain individuals. For example, Abi‐Melhem and Hassoun (2023) described six cases of pea allergy, five of which involved children under 10 years old, and a 15‐year‐old boy with a history of cashew allergy but tolerance to peanut. The latter immediately developed hives on his face and neck in three incidences after consumption of processed food that contained green peas, such as substitute milk, substitute chicken nuggets, and pea‐based protein powder. The severe response was accompanied by an elevated IgE level to pea extract. Another report described a 28‐year‐old woman with asthma who had a severe reaction to falafel containing peas (Dölle‐Bierke et al. 2022). She had a similar reaction to peas when she was 10 years old and has since been cautiously avoiding peas and peanuts. Her skin prick test (SPT) was positive, and specific IgE to pea was slightly increased. However, other legumes, nuts and sesame showed negative in the SPT. These cases raised a question whether peas are safe for people with sensitization to homologous allergens although the allergenic reactions were reported to be due to cross reactivity, and in particular, due to pea vicilin‐like protein‐induced cross reactivity. Cohort data from Italy demonstrated that pea LTP (Pis s 3) is safe to consume for LTP‐allergic patients (Alessandri et al. 2021), whilst a German cohort study discovered the seed storage protein Pis s 1 as a major immunodominant allergen (Popp et al. 2020).

#### 3.1.5 Lentils

Lentils ( *Lens culinaris* ) are a rich source of protein with antioxidant and anti‐inflammatory benefits (Joehnke et al. 2021; Mustafa et al. 2022). The lentils' unique flavor has led to their widespread use in vegan products (Präger et al. 2023; Reese et al. 2023). In addition, lentils' proteins can induce allergic reactions through numerous routes of exposure such as ingestion, inhalation, and skin contact. Several unique case studies highlighted the potential allergenic risk of lentils. For example, a 9‐year‐old boy developed anaphylaxis after consuming lentil soup. Following this incident, the boy removed lentils from his diet. However, during a separate incident involving direct skin contact with lentil soup, he experienced itching, redness, and swelling of the skin (Akpınar 2018). Another example involves a 22‐months‐old girl who developed an allergic reaction shortly after inhaling the vapors from cooking lentils. A positive specific IgE signal for lentils confirmed the lentil sensitization (Vitaliti et al. 2012). More recently, a rare case of FDEIA after eating lentils was published by Alnabulsi et al. (2023) where a 16‐year‐old girl experienced respiratory distress with urticaria‐like symptoms upon exercising within an hour after eating lentils. These symptoms recurred again under similar circumstances. Interestingly, her IgE levels for lentils were low. Overall, the cases collectively highlight that lentil allergy can be multi‐route, IgE‐driven, and potentially severe, with variable clinical presentation depending on exposure context and co‐factors.

#### 3.1.6 Chickpea

Chickpeas ( *Cicer arietinum* ) are among the most frequently eaten legumes and are used in a variety of popular vegan recipes such as falafel and hummus. Despite the low incidence of chickpea allergy, some old reports back to 2008 have documented cases of FDEIA related to eating chickpea. For instance, a 16‐year‐old boy (Orhan and Karakas 2008) and a 17‐year‐old girl (Roberts and Ben‐Shoshan 2015) experienced anaphylaxis after eating chickpeas and performing subsequent exercise. Both showed SPT positive results for chickpea, whilst IgE was only determined for the latter, indicating high IgE titers for chickpea and soybean (Roberts and Ben‐Shoshan 2015). These cases point out the potential allergenic risk of chickpeas and the need for further research. Overall, limited recent data on chickpea allergy exists.

#### 3.1.7 Lupine

*Lupinus species* exhibit similar nutritional value as other legumes such as peas and lentils. However, they are used as foods in addition to being used as a food ingredient (Cabello Hurtado et al. 2016). The most used lupine species include *L. angustifolius* , *L. albus* , and *L. luteus* while *L. mutabilis* is only popular in South America (Jappe and Vieths 2010). Lupine seeds and/or their respective flours are used as nutritional substitutes for people with allergies or disorders such as animal (milk, eggs) and plant‐based foods (gluten, wheat) respectively (Hefle et al. 1994). Lupine allergy was first reported in the United States (Hefle et al. 1994), then in Germany (Brennecke et al. 2007) and recently in a Canadian child with peanut allergy (Soller et al. 2018). Various researchers (Jappe and Vieths 2010; Skypala 2019; Jardim Botelho et al. 2022) described lupine allergens as “hidden allergens” due to numerous factors including undeclared or unexpected use in processed foods, cross‐contamination and substitution with other flours, incomplete labeling awareness, and lower consumer familiarity.

Allergy to lupine manifests as single allergen allergy or due to the protein cross‐reactivity of different legumes, with peanut the most to exhibit cross reactivity (Jappe and Vieths 2010). The patterns of clinical cross‐reactivity that occur most frequently among legumes include peanut, lupine, soy, chickpea, and lentil, and this is influenced by both regional variation and frequency of these foods in the diet (Chan et al. 2019). The WHO/IUIS Allergen Nomenclature sub‐committee has only acknowledged three allergens including Lup an 1 (a β‐conglutin of *L. angustifolius* ), Lup an 3, a non‐specific LTP of *L. angustifolius* , and Lup a 5, the profilin of *L. albus* , thanks to allergen composition and structure across different lupine species (Jappe et al. 2021). According to Guillamón et al. (2010), white lupin is a protein‐rich legume with high lysine content and valuable functional, hypocholesterolemic, and hypoglycemic properties. Its major storage proteins (β‐ and α‐conglutins) are also major allergens with potential cross‐reactivity to peanut proteins.

#### 3.1.8 Cowpea

Cowpea ( *Vigna unguiculata* ) is among the non‐woody plants and grows annually. It is characterized by its resistance to drought (Omomowo and Babalola 2021). Chentouh et al. (2022) demonstrated that a large number of legume allergy illnesses was associated with cowpea proteins in Luxembourg. The study illustrated four allergens with potential protein cross‐reactivity to other legumes. When utilizing crystallographic characterization, Chanana et al. (2004) found a potential allergen from the 2S albumin family in cowpea (25 kDa). The 2S albumin family is highly resistant to digestion, and it interacts with membranes, both of which are critical factors responsible for food allergenicity.

Collectively, a major characteristic of legume allergy is the structural homology shared among key seed storage proteins. For example, vicilins (7S globulins), legumins (11S globulins), and 2S albumins possess conserved amino acid sequences and three‐dimensional conformations across many legume species, resulting in shared IgE‐binding epitopes that contribute to immunological cross reactivity. Specifically, homologous vicilin anf legumin proteins are present in peanut, soybean, pea, lentil, chickpea, and lupin, explaining why the sensitization to one legume may lead to IgE recognition of another. Nevertheless, structural similarity alone doesn't always predict clinical allergy, since allergenic responses are also influenced by epitope accessibility, protein stability during digestion, food matrix effect, and patient‐specific immune responses. These mechanistic differences are particularly relevant when evaluating non‐thermal technologies as their effectiveness depends on their ability to modify conserved structural epitopes shared across homologous allergens.

## 4 Application of Non‐Thermal Technologies in Modulating Legumes Allergy

NTTs represent advanced food processing methods that inactivate microorganisms and alter food components without the use of high temperatures, thereby maintaining the nutritional and sensory integrity of the product (Dangal et al. 2024; Shinde et al. 2024; Kulawik et al. 2023). These innovative approaches address the limitations of traditional thermal processing by providing reliable alternative solutions (Barba et al. 2018; Misra et al. 2017). NTTs offer distinct benefits, including the preservation of organoleptic, nutritional, and bioactive attributes (such as antioxidants and phenolics), along with the reduction of immunoreactivity in foods like eggs, soybeans, and peanuts (Filep and Chapman 2022; Zhao et al. 2022).

NTTs are grouped into physical, radiative/electromagnetic, mechanic, chemical, and combined categories, with all exhibiting the capability to achieve microbial safety and shelf stable products while also preserving sensory and nutritional quality (Barbhuiya et al. 2021; Masilamani et al. 2025). Table 2 illustrates the classifications of NTTs, with technologies such as pulsed electric field (PEF), high‐pressure processing (HPP), ionizing radiation (IOR), ultraviolet (UV) light, pulsed light (PL), and cold plasma (CP) falling into 1 or more of these categories. They are all increasingly being adopted for preserving heat‐sensitive foods due to their efficacy in microbial inactivation without significant thermal input, thus minimizing the degradation of bioactive compounds (Al‐Sharify et al. 2025). In contrast to conventional thermal processes that may compromise food quality, non‐thermal processes retain the texture, taste, and rheological properties; therefore, presenting an invaluable option in the food chain system (Al‐Sharify et al. 2025; Dangal et al. 2024). Moreover, non‐thermal food processing approaches are becoming well‐established within the food sector, as they can be utilized for a wide range of food items (Al‐Sharify et al. 2025).

**TABLE 2:** Classifications of NTTs.

No	Category	Examples of NTTs	Mechanism of action	Application areas	References
1	Physical methods	High‐pressure processing (HPP), Pulsed electric fields (PEF), Ultrasound (US), Cold plasma (CP)	Disruption of cell membranes, protein denaturation, mechanical and electrical effects	Juices, dairy, meat, and ready‐to‐eat foods	Jadhav et al. (2021)
2	Electromagnetic/radiative methods	Ultraviolet (UV‐C) light, Ionizing radiation (gamma, X‐ray, electron beam), Infrared (IR)	DNA damage, oxidative stress, photochemical reactions	Surface sterilization, liquid foods, spices	Zhang and Wang (2019)
3	Mechanical/acoustic methods	Ultrasound (low and high frequency), Hydrodynamic cavitation	Shear forces and cavitation cause cell disruption	Emulsification, microbial inactivation, extraction	Ojha et al. (2020)
4	Chemical‐assisted non‐thermal methods	Ozone treatment, Supercritical CO_2_, Dense phase CO_2_, Plasma‐activated water	Oxidation and disruption of microbial structures, pH modification	Juices, seafood, fresh produce	Shi et al. (2021)
5	Combined (hurdle) approaches	PEF + mild heat, HPP + antimicrobials, Ultrasound + UV	Synergistic effects enhancing safety and quality	Preservation of minimally processed foods	Masilamani et al. (2025)

Recent research suggests that novel processing technologies may reduce the allergenicity of specific proteins and induce changes in protein three‐dimensional shape while retaining the food properties (Dong et al. 2021). For example, ultrasound treatment may lower allergenicity by altering the microstructure of kiwifruit tissue (Yuan et al. 2021). Likewise, Pulsed light (PL) is known for its ability to modify the molecular structure of proteins or aggregated dietary proteins and act by altering the structure of allergenic proteins without significant heat exposure (Alhendi 2021). Other studies by Zahid et al. (2025) and Zhong and Xie (2019) respectively highlighted the ability of NTTs to modify and alter the structural and functional properties of allergenic proteins with less degradation while HPP can unfold protein structures, leading to the exposure or masking of epitopes involved in immune recognition, thereby reducing allergenicity. Together, these findings highlight the potential of NTTs as targeted tools for allergenicity modulation and set the stage for a closer examination of individual non‐thermal technologies and their specific mechanisms of action in the following section.

### 4.1 High‐Pressure Processing (HPP)

**FIGURE 1:**
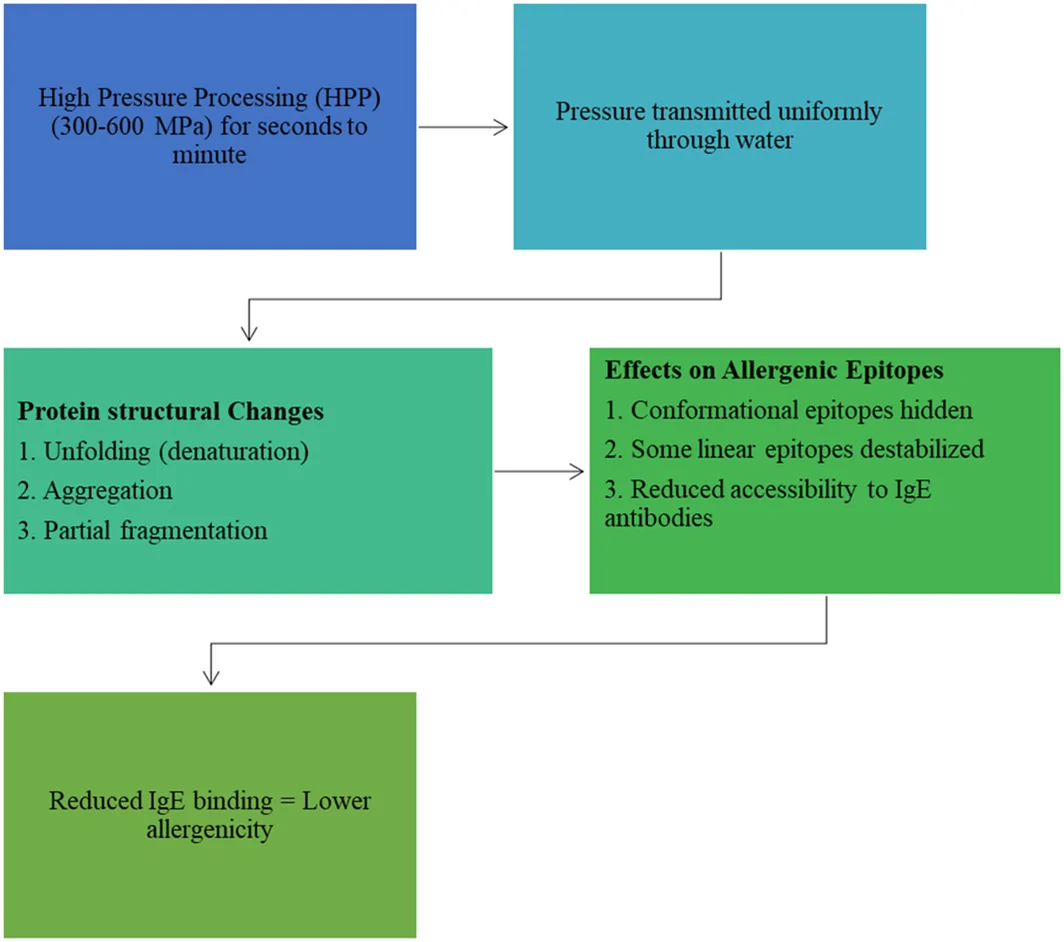
Mechanisms of high‐pressure processing (HPP) in legume allergenicity reduction (adapted from Lavilla et al. 2020).

HPP, a non‐thermal technology with pressure ranging from 100 to 800 MPa, is widely used in the food industry (Abera 2019). It enables the modification of food carbohydrate and protein functionalities, sterilization, micronutrient preservation, inactivation, and activation of endogenous enzymes (Asaithambi et al. 2021; Dehnad et al. 2024). In HPP, proteins undergo an irreversible or reversible structural modification that results in aggregation, gel formation, and denaturation (Costa et al. 2022). As reported by Huang, Hsu, et al. (2014) and Huang, Yang, and Wang (2014), HPP reduces the allergenicity of food proteins in various ways such as by releasing allergenic proteins through permeating the surrounding solution, solubilizing proteins, or diffusing solubilized proteins. When applied at elevated pressure, HPP shows a considerable impact in reducing the immunoreactivity of food by aggregation and protein denaturation (Gharbi and Labbafi 2018). In addition, a combination of HPP with other techniques such as enzymatic hydrolysis or PEFs was reported to improve the reduction in allergenicity (Pang et al. 2024). Different levels of HPP were reported to potentially affect soy protein content. For example, when applied at 600 MPa for 5 min, it resulted in a significant increase in the protein content of soymilk whereas at 300 MPa (5 min), a reduced soy Kunitz trypsin inhibitor (SKTI) antigen binding ability was reported (Rajan et al. 2023). This suggests breakage of particle matrices, allowing the entrapped proteins to interact with surrounding liquid suspension. Figure 1 illustrates the mechanisms of high‐pressure processing (HPP) in legume allergenicity reduction.

### 4.2 Pulsed Electric Fields (PEF)

**FIGURE 2:**
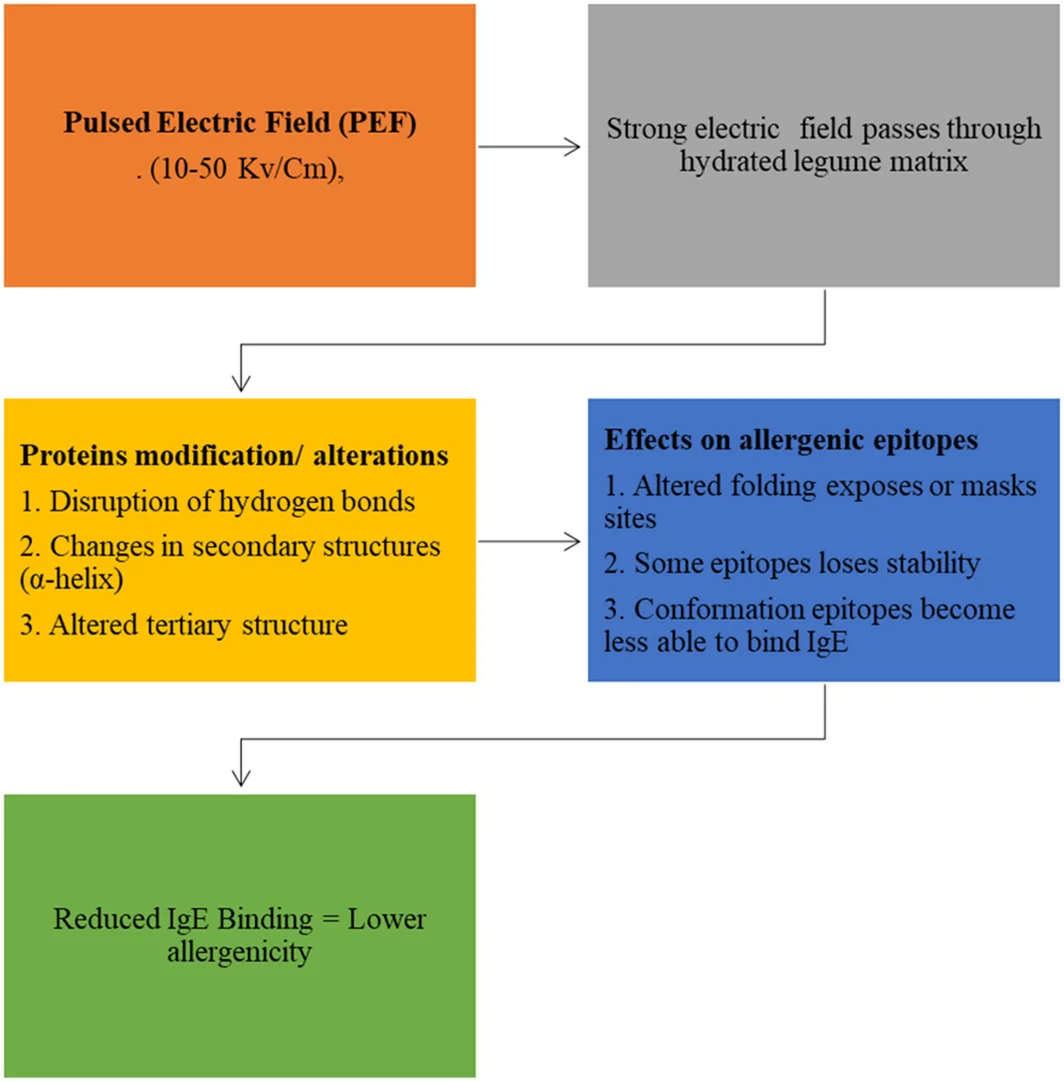
Mechanisms of pulsed electric field (PEF) in legume allergenicity reduction (adapted from Shah et al. 2019).

Pulsed Electric Field (PEF) is a novel technique that uses brief bursts of high‐intensity electric fields (ranging from 0.1 to 80 kV/cm) applied for durations from nanoseconds to milliseconds (Xiao et al. 2025). The food is positioned between two electrodes, allowing the treatment to disrupt and increase the permeability of microbial cell membranes while preserving or enhancing the food's quality characteristics (Bhat et al. 2019; Giancaterino et al. 2023). After allergen mitigation, PEF treatment can induce structural modifications in allergenic proteins. Figure 2 shows the mechanisms of pulsed electric field (PEF) in legume allergenicity reduction. In soybean, PEF treatment significantly reduced the allergenicity of glycinin and β‐conglycinin by modifying protein conformation and digestion‐resistant linear epitopes, with electric field strengths of 20–30 kV/cm producing the greatest effect. Although studies on other legumes remain limited, current evidence suggests that PEF has considerable potential for producing hypoallergenic legume‐based foods while preserving their nutritional and functional properties (Ding et al. 2025; Ramaswamy and Krishnan 2024). However, contradictory findings on the PEF ability to alter protein conformational structure were also reported (Johnson et al. 2010). In their study, the authors evaluated the PEF treatment on native Ara h 2, 6 and concluded that this treatment doesn't induce any significant changes in the structure of any of the allergens evaluated. The above contradictory findings necessitate further studies on the efficacy of PEF treatment to evaluate whether its ability to modify protein structures depends on particular legume species.

### 4.3 Cold Plasma (CP)

**FIGURE 3:**
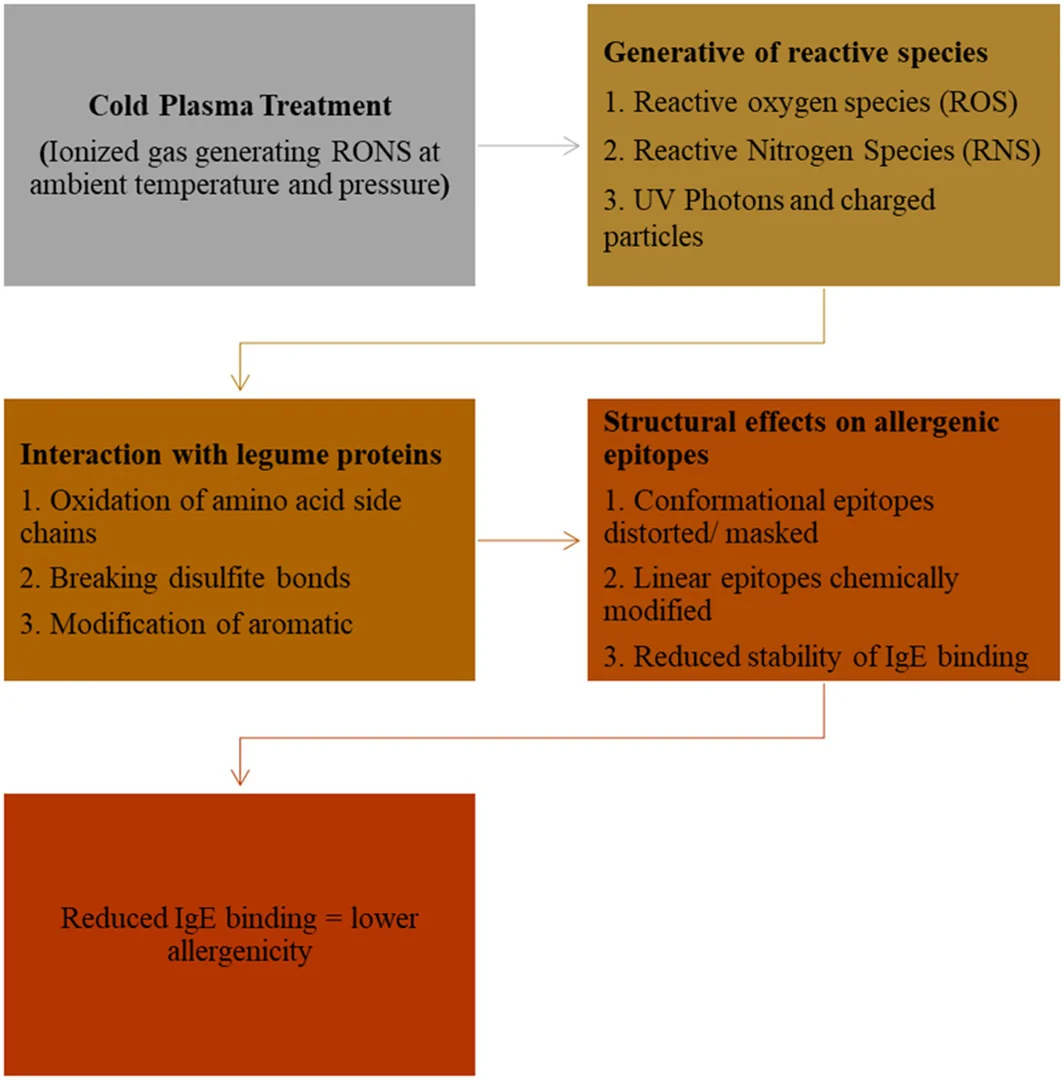
Mechanisms of cold plasma (CP) in legume allergenicity reduction (adapted from Meinlschmidt et al. 2016 and Venkataratnam et al. 2019).

Cold plasma (CP) is a food processing technique known for enhancing food safety through effective microbial decontamination and prolonging the shelf life of products (Gavahian and Khaneghah 2020). It is considered an environmentally friendly and sustainable technology because it generates no chemical or toxic residual substances in the food after processing and operates with low energy consumption (Tabares and Junkar 2021). Key components of a CP processing system include a ceramic electrode and a plasma generator (Ekezie et al. 2017). Plasma, an ionized gas, consists of a variety of reactive species around numerous different types including electrons, molecules, ultraviolet photons, free radicals, ions, and excited atoms, which form when the gas becomes energized and ionized (Gavahian and Khaneghah 2020). Reactive species generated during plasma treatment can interact with food proteins, leading to alterations in their three‐dimensional structures (Tolouie et al. 2018). These plasma‐induced modifications to both conformational and linear epitopes contribute to the inactivation of allergens during plasma processing (Meinlschmidt et al. 2016). Additionally, reactive species may compromise protein stability by oxidizing amino acid residues and breaking peptide bonds. For example, hydroxyl radicals can cleave disulfide bonds in peptides, resulting in the formation of RSH and RSO groups at the cleavage sites. Reactive nitrogen and oxygen species also target oxidizable amino acids, potentially disrupting antibody binding sites (Gavahian and Khaneghah 2020; Tolouie et al. 2018; Li et al. 2017). The effectiveness of allergen reduction via plasma is influenced by several factors such as the type of plasma source, the gas used, treatment duration, and the nature of the substrate, all of which affect the production and penetration of reactive species. Figure 3 summarizes the mechanisms of cold plasma (CP) in legume allergenicity reduction.

Venkataratnam et al. (2020) evaluated the impact of CP treatment on key peanut allergens, Ara h 1 and Ara h 2. In their experiment, the high‐voltage power supply used to generate the plasma discharge in atmospheric air was set to a resonant frequency of 52 kHz and a discharge voltage of 32 kV, and the duty cycle was maintained at 118 s with a discharge frequency of 1 kHz. Sodium Dodecyl Sulfate‐Polyacrylamide Gel Electrophoresis (SDS‐PAGE) analysis indicated that increasing plasma exposure from 15 to approximately 60 min led to a reduction in the solubility of these allergenic proteins. When purified from defatted peanut flour (DPF), the antigenicity of Ara h 1 and Ara h 2 decreased significantly by 65% and 66%, respectively. In a related study, Venkataratnam et al. (2019) investigated the antigenicity of Ara h 1 under cold atmospheric plasma treatment. Dry, DPF, and whole peanuts were subjected to an 80 kV plasma at different time intervals (0, 15, 30, 45, and 60 min). SDS‐PAGE results showed no significant change in the intensity of Ara h 1 bands for both whole peanuts and DPF. However, competitive ELISA tests revealed antigenicity reductions of up to 9.3% for whole peanuts and 43% for DPF. These findings suggest that CP treatment can effectively reduce Ara h 1 antigenicity, accompanied by lasting changes in protein structure. Zhang et al. (2021) studied the effects of atmospheric cold plasma (ACP) on soybean protein isolate (SPI), finding that ACP‐induced reactive oxygen species caused oxidation that altered SPI's secondary and tertiary structures. Moderate ACP treatment of 120 Hz applied frequency for 5 min enhanced functional properties like emulsifying and foaming while reducing allergenicity by up to 75% in IgE binding; however, excessive exposure led to protein aggregation and decreased functionality.

### 4.4 Ultrasound (US)

**FIGURE 4:**
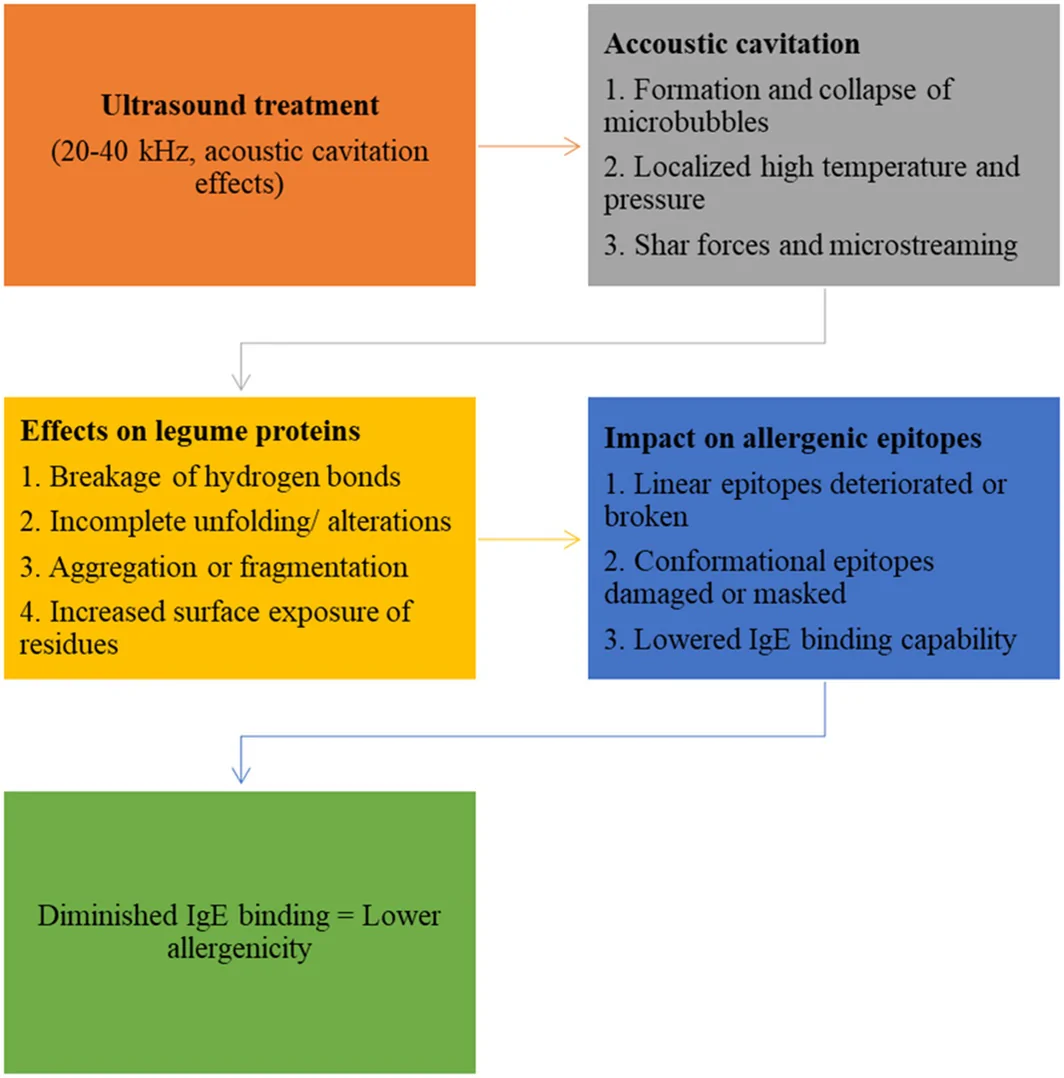
Mechanisms of ultrasound (US) in legume allergenicity reduction (adapted from Yang et al. 2015).

Ultrasound refers to sound waves with frequencies above the human hearing range (typically uses frequencies from 20 kHz to several MHz) and high intensity (typically over 1 W/cm^2^) (Cavallo et al. 2020). Ultrasonication (US) involves applying these high‐frequency waves in a liquid, causing cavitation, the formation, growth, and collapse of gas bubbles due to pressure changes (Ashraf et al. 2023; Ekaette and Saldaña 2021). This technology offers advantages like lower processing temperatures and times, enhanced mass and energy transfer, reduced costs, and efficient energy use (Vanga et al. 2020). It is widely applied in food processing for tasks such as tenderization, sterilization, drying, enzyme inactivation, emulsification, and extraction (Cavallo et al. 2020; Raza et al. 2023). US treatment has been shown to reduce allergenicity in various foods; Figure 4 demonstrates the mechanisms of ultrasound (US) in legume allergenicity reduction. Yu et al. (2016) found that ultrasound‐assisted germination of peanut sprouts elevated resveratrol levels from 1.71 to 3.34 times the initial content while lowering allergenic potential by 11.0 to 76.4% depending on peanut cultivar. Similarly, Yang et al. (2015) reported decreased IgE‐binding capacity in sprouted soybean proteins after ultrasound pretreatment, likely due to epitope disruption.

### 4.5 Enzymatic Hydrolysis

Enzymatic hydrolysis is extensively acknowledged as a highly effective, selective, and ecologically sustainable methodology for the synthesis of hypoallergenic food products (Zhang et al. 2018). This technique utilizes proteolytic enzymes to cleave peptide bonds, thereby disrupting both linear and conformational epitopes that bind immunoglobulin E (IgE). By degrading proteins into smaller peptides and amino acids, enzymatic treatment can markedly diminish immunoreactivity (Ekezie et al. 2018). Traditionally employed in the formulation of hydrolyzed infant formulas (Larson‐Nath et al. 2025), this technology has broadened its application to encompass a variety of allergenic food substances. For instance, combined trypsin and α‐chymotrypsin treatments have significantly decreased the immunoreactivity of peanut allergens Ara h 1 and Ara h 2 (Yu et al. 2013). Comparable reductions in immunoreactivity have been documented for legume protein hydrolysates such as those derived from kidney beans, black gram, and peanuts with 62.2%, 87.1%, and 91.8% reductions respectively (Kasera et al. 2015).

Importantly, enzymatic hydrolysis can be synergistically combined with other non‐thermal methodologies, such as high‐pressure processing or ultrasound, to enhance enzyme accessibility and facilitate structural unfolding without the deleterious effects of thermal exposure. Such integrative approaches enhance epitope exposure and proteolytic efficacy, thereby offering a promising avenue for modulating legume allergenicity while safeguarding nutritional integrity.

### 4.6 Combination of Processing Technologies

Combining processing technologies has shown enhanced potential for reducing food allergenicity. For instance, Zhou et al. (2016) reported that HHP could significantly improve the extent of proteolysis by papain, alcalase, or pepsin and reduce the antigenicity of gingko seed proteins. Amponsah and Nayak (2016) reported that microwave and ultrasound‐assisted extraction of soy proteins influenced protein composition, notably glycinin and β‐conglycinin. Similarly, Meinlschmidt et al. (2016) demonstrated that combining gamma irradiation, pulsed UV light, and CP achieved up to 91%–100% reduction in soy protein immunoreactivity, suggesting the great potential of the technologies in industrial applications due to their observed effectiveness to reduce soy immunoreactivity. Further, combining HPP with enzymatic hydrolysis enhanced epitope disruption and allergen breakdown (Meinlschmidt et al. 2017). Collectively, these studies underscore the potential of combined physical treatments to reduce food allergenicity effectively, though careful processing parameter optimization is crucial to preserve product quality as previously stated.

## 5 Mechanistic Insights Into Allergenicity Modulation by NTTs

Reducing the allergenic potential of proteins that cause allergic reactions is essential for tackling the increasing prevalence of allergies worldwide. Proteins are known to undergo structural heat‐induced changes which in turn affect their functionality including solubility, emulsion stability, and bioactivities. Aside from heat, Wei et al. (2025) highlighted that protein dimensional structure, coagulation properties, and protein solubility are impacted by non‐thermal methods including HPP and PEF. Qian et al. (2023) revealed that US modifies protein structure which facilitates the breakdown of long peptide chains and hence the improved digestibility. Tan et al. (2024) elucidated that the application of CP resulted in an augmentation of the surface hydrophobicity of soybean protein isolates concomitant with an escalation in treatment duration. Increased surface hydrophobicity may expose previously buried hydrophobic epitopes or, conversely, promote aggregation that masks epitopes. This phenomenon may be attributed to the oxidative modification that induces unfolding of the protein structure, thereby revealing hydrophobic groups that are ordinarily sequestered at the interfaces of protein domains and subdomains, or at the junctions of subunits within oligomeric protein assemblies, consequently enhancing the accessibility of sodium 8‐anilino‐1‐naphthalenesulfonic acid (ANS‐N).

The utilization of CP resulted in modifications to the secondary structure, evidenced by an enhancement in the aggregated β‐sheet conformation alongside a simultaneous decrease in the random coil structure of proteins, indicating that aggregation transpires following the unfolding of proteins as a result of CP exposure (Horta et al. 2026). The implementation of CP on ovine milk led to significant alterations in the secondary conformational structure of proteins, effectively inhibiting protein aggregation while simultaneously promoting the formation of β‐sheet structures. The alterations in protein structure are also associated with a reduction in allergenicity (Ekezie et al. 2017). Table 3 illustrates the immunological mechanisms of food allergens. Allergenicity is reduced by changing protein structures so IgE antibodies cannot bind and trigger allergic reactions (Mills et al. 2009).

**TABLE 3:** Immunological mechanisms of food allergens.

Process	Key mechanism	Mechanistic description	Immunological outcome	References
Barrier protection damage and promoting antigen contact	Malfunction of epithelial barrier such for skin, and gut	Tight‐junction damage, filaggrin mutations, and inflammation allow larger food proteins to cross epithelial surfaces	Enhanced allergen permeability which leads to high exposure of antigen‐presenting cells (APCs)	Johnston et al. (2014)
	Non‐oral routes of sensitization (skin/airway)	Allergen exposure through eczematous skin or inhalation instead of oral route	Promote IgE sensitization without tolerance induction; common in early childhood atopy	Chinthrajah et al. (2016)
	Microbiome dysbiosis	Reduced microbial diversity and SCFA‐producing bacteria	Impaired Treg induction which lead to diminished tolerance and increased Th2 bias	Chinthrajah et al. (2016)
Antigen recognition and antigen‐presenting cells (APCs)	DCs macrophages capture food‐antigens	Intestinal DCs such as CD103* present allergenic peptides to naïve T cells	Activation and polarization of naïve T cells toward a Th2 phenotype, promoting IL‐4, IL‐5, and IL‐13 production and initiating IgE sensitization	Chinthrajah et al. (2016)
T‐cell responses	Regulatory T‐cells (Tregs) and tolerance	In non‐allergic states, Tregs suppress responses to food antigens, maintain IgA production, promote homeostasis	Maintenance of oral tolerance by suppressing Th2 responses and limiting IgE production. Impaired Tregs induction of function contributes to the loss of oral tolerance and the development of food allergy.	Chinthrajah et al. (2016)

The destruction of epitopes entails the breakdown of chemical bonds within the protein matrix, particularly affecting linear epitopes, resulting in their degradation and subsequent inability to interact with IgE antibodies (Mills et al. 2009). These bonds have been reported to be non‐covalent in nature, including hydrogen bonds, hydrophobic interactions, and disulfide bonds. The masking of epitopes requires structural alterations, such as protein denaturation and aggregation, which can effectively obscure conformational epitopes, rendering them inaccessible to the antibodies of the immune system (Verhoeckx et al. 2015). NTTs provide a mechanism for reducing allergenicity through the inactivation or modification of conformational epitopes. For instance, HHP conceals conformational epitopes due to the elevated pressure applied, which leads to the denaturation of proteins and promotes aggregation (Lavilla et al. 2016). This principle was reported in soybeans and shrimp proteins allergenicity reduction (Huang, Hsu, et al. 2014; Huang, Yang, and Wang 2014). PEF, a NNT which uses high voltage pulses, causes change in protein secondary and tertiary structures, affecting the accessibility of conformational epitopes (Li et al. 2022). US treatment was reported to cause both physical and chemical changes that can lead to protein degradation and the destruction or modifications of epitopes (Hu et al. 2014). CP was reported to generate both reactive nitrogen and oxygen species that react with protein antigens, altering their structure and antibody binding sites (Afshari et al. 2020).

IgE‐binding capacity is a critical determinant of allergenic potential, as hypersensitivity reactions are initiated when immune cells recognize allergenic proteins. Yu et al. (2020) evaluated the inhibitory effect of alginate, a soluble dietary fiber isolate from *Laminaria japonica* (a brown algae) on allergenic reactions in an ovalbumin‐induced mouse model. Alginate was efficient in lowering sensitization by weakening the allergenic protein absorption through encapsulation. Table 4 is the summary of in vitro and in vivo studies supporting hypoallergenic potentials of legumes. Overall, both thermal and non‐thermal processing methods can reduce legume allergenicity through several mechanistic pathways, though effectiveness varies with species, allergen type, and processing conditions. Extrusion and fermentation are among the most effective single treatments, while non‐thermal technologies such as HPP, CP, PEF, US, and enzymatic hydrolysis often achieve substantial reductions in IgE‐binding with minimal nutritional loss. Greater allergenicity reduction is generally obtained through combined or sequential treatments, particularly when physical processing is coupled with enzymatic hydrolysis; however, no approach ensures complete hypoallergenicity, and clinical validation remains limited.

**TABLE 4:** Summaries of in vitro and in vivo studies supporting hypoallergenic potential of legumes.

No.	Study	Method	Findings	References
1	The inhibitory activity of alginate against allergic reactions in ovalbumin‐induced mouse model	The alginate isolate obtained from brown algae, *Laminaria japonica*, were administered in Ova‐induced BALB/c mice (mouse model allergy)	The study revealed the alginate potential antiallergenic activity in mouse model of egg allergy	Yu et al. (2020)
2	Peanut protein allergens: gastric digestion is carried exclusively by pepsin	In vitro gastric digestion assay simulating stomach condition to evaluate stability of major peanut allergens including Ara h1, Ara h2, and Ara h3.	Pepsin was found as the responsible enzyme for gastric digestion of peanut proteins under acidic conditions. Some peanuts allergy specifically Ara h2 digestion exhibited partial resistance to pepsin digestion	Kopper et al. (2004)
3	Effectiveness of different proteases in reducing allergen content and IgE‐binding of raw peanuts	Raw peanut kernels were treated with four food enzymes including Alcalase, papain, neutrase, bromelain; Allergen residues (Ara h1, Ara h2, and Ara h6) were quantified using Sandwich ELISA and SDS‐PAGE while IgE‐binding was evaluated via Western blot	All four proteases were effective to reduce Ara h1. A substantial reduction of Ara h2 and Ara h6 was noted. 100% and 99.8% reductions of Ara h1, Ara h2 and Ara h6 were achieved respectively with alcalase. Overall, alcalase was found to be the most effective of the four evaluated proteases. Bromelain and Neutrase were comparatively less effective for Ara h2 and Ara h6. Hydrolysis also produced smaller peptides with strong IgE‐binding.	Yu and Mikiashvili (2020)
4	In vitro digestibility and immunoreactivity of thermally processed peanut	Thermal Processing: Baisha peanuts were processed by either roasting (20 min at 160°C in an oven) or boiling (20 min in water), with raw peanut used as a control Digestion Simulation: Samples were subjected sequentially to three phases Oral Phase: Simulated Salivary Fluid (SSF) containing amylase and lysozyme Oral‐Gastric Phase: Simulated Gastric Fluid (SGF) containing pepsin (2000 U/mL), incubated at 37°C for various time points, up to 120 min Intestinal Phase: Gastric digests (10 min samples) were further treated with Simulated Intestinal Fluid (SIF) containing trypsin, chymotrypsin, amylase, lipase, and colipase, incubated for up to 60 min. Boiling significantly enhanced the overall digestibility and reduced the IgE‐binding capacity of the resulting digestion products, whereas digestion‐resistant fragments of key allergens like Ara h 2/6 still persisted across all treatment groups	Peanut proteins were rapidly digested by pepsin, with digestion evident after only 2 min, characterized by a loss of high molecular weight proteins and an enrichment of polypeptides lower than 10 kDa Ara h 1 and Ara h 3 were partly proteolyzed Ara h 2/6 contained a digestion‐resistant fragment and was poorly digested, still being present in the soluble fraction after 120 min of gastric digestion in all processed peanuts Numerous digestion‐resistant fragments belonging to Ara h 2/6 remained even after intestinal digestion Ara h 3 isoforms were still identified in raw and roasted peanuts after 120 min of digestion	Rao et al. (2018)

A major challenge in comparing the effectiveness of NTTs is the wide variation in processing parameters and conditions, including pressure, the strength of the electric field, treatment duration, temperature, gas composition, and food matrix. Instead of applying universal treatment conditions, future studies should adopt harmonized reporting standards that consider complete processing parameters such as pressure/electric field strength, treatment time, temperature, pulse characteristics, or gas composition where applicable; detailed sample characteristics; standardized allergenicity assessment methods; and evaluation of both allergenicity reduction and functional food quality. According to the available literature, moderate to high processing intensities generally produce greater reductions in IgE binding, although excessive treatments may promote protein aggregation or adversely affect functional properties. Consequently, optimization should balance allergenicity reduction with nutritional and technological quality using response surface methodology or similar experimental designs. Table 5 is the summary of studies comparing NTTs in legumes and illustrates their effects on various legume allergens.

**TABLE 5:** Summary of studies comparing non‐thermal technologies (NTTs) in legumes.

No.	Studies	Processing method	Legumes types	Key findings	References
1	Effect of processing treatments on the allergenicity of nuts and legumes: A meta‐analysis	Single extrusion, and fermentation; combined fermentation and proteolytic hydrolysis	Various nuts and legumes (mixed dataset of 61 in vitro studies)	Among single treatments, extrusion and fermentation showed the largest reduction in allergenicity. The combined treatment (fermentation followed by proteolytic hydrolysis) had the greatest reduction	Astuti et al. (2023)
2	Effect of thermal processing and γ‐irradiation on allergenicity of legume proteins	Boiling (thermal), γ‐irradiation alone, combined boiling and γ‐irradiation	Kidney bean, black gram, peanut	Boiling reduced soluble‐protein IgE‐binding by 74% ± 6.5% (kidney bean), 83% ± 11.6% (black gram), 62% ± 7.2% (peanut). Boiling followed by γ‐irradiation further decreased IgE binding (potency of soluble proteins reduced up to 26‐fold; insoluble up to 8‐fold). γ‐irradiation alone was ineffective.	Kasera et al. (2012)
3	Effect of processing on soybean allergens and their allergenicity	Thermal processing; fermentation; enzymatic degradation; sprouting; combination methods	Soybean	Thermal processing, enzymatic degradation, sprouting and fermentation were reported to reduce soybean allergenicity by destroying epitopes or reducing major allergen proteins. Some protein allergens stable to heat/sprouting were degraded by enzymatic degradation or fermentation	Pi et al. (2021)
4	Non‐Thermal Food Processing for Plant Protein Allergenicity Reduction: A Systematic Review	Non‐thermal: high‐pressure processing (HPP), cold plasma, ultrasound, pulsed electric fields (PEF), enzymatic digestion, bacterial fermentation	Plant proteins (various legumes, nuts)	Non‐thermal processing technologies have potential to reduce plant‐protein immunoreactivity by > 50% compared to untreated controls (under adequate conditions). Allergenicity reduction seems primarily via structural modifications (secondary/tertiary structure), potentially disrupting IgE‐binding epitopes.	Arteaga‐Marin et al. (2025)
5	Effectiveness of enzymatic hydrolysis for reducing the allergenicity of legume by‐products	Enzymatic hydrolysis (protease: alcalase or papain)	Chickpea, green pea, white bean by‐products	Alcalase hydrolysates of chickpea and green pea showed no residual immunoreactivity toward sera from legume‐allergic patients. Papain hydrolysates retained some immunoreactivity. For white bean, even alcalase hydrolysis left residual immunoreactivity, likely due to antinutritional factors (lectins/phytohemagglutinins) that resisted hydrolysis.	Calcinai et al. (2022)
6	Mitigation of peanut allergenic reactivity by combined processing: Pressured heating and enzymatic hydrolysis	Pressured heating + enzymatic hydrolysis	Peanut (major peanut allergens, including ns‐LTP)	Heat/pressure treatment reduced IgE‐binding; subsequent enzymatic hydrolysis markedly reduced allergenic potency, especially for non‐specific lipid transfer protein (nsLTP) peanut allergens. Combined treatment was more effective than either alone	Cuadrado et al. (2023)

### 5.1 Factors Influencing the Effectiveness of NTTs

The effectiveness of non‐thermal technologies is highly associated with the inherent characteristic of individual legume allergens. Protein structural stability, molecular size, disulfide bond content, hydrophobicity, and relative abundance of conformational versus linear IgE‐binding epitopes have an influence on the susceptibility to structural modification during processing (Mills et al. 2009; Verhoeckx et al. 2015). In addition, food matrix composition, moisture content, pH, ionic strength, and interactions with carbohydrates or lipids can alter protein unfolding and epitope accessibility, resulting in differences in allergenicity reduction among legumes processed under similar conditions (Verhoeckx et al. 2015). Consequently, future optimization should adopt allergen‐specific instead of focusing on technology‐specific approaches by integrating structural protein characterization with processing design to identify the most effective NTT for each legume species.

### 5.2 Influence of the Food Matrix and Gastrointestinal Digestion

Apart from the structural modifications driven by NTTs, the allergenicity of processed legumes is also affected by their interactions between proteins and other food components such as carbohydrates, fibers, fats, and polyphenols, which can impact protein solubility, aggregation behavior, epitope accessibility, and enzyme susceptibility, thereby influencing the immunological response after ingestion (Mackie et al. 2012; Verhoeckx et al. 2015). During gastrointestinal digestion, NTT‐induced unfolding may increase protease accessibility and promote degradation of allergenic epitopes; however, excessive aggregation or oxidation may reduce enzyme accessibility, leading to the persistence of digestion‐resistant peptide fragments that retain IgE‐binding capacity (Mills et al. 2009). Importantly, assessment of NTT‐treated legumes should extend beyond structural analyses to include simulated gastrointestinal digestion and bioaccessibility studies in a realistic food matrix to better predict in vivo allergenic potential.

## 6 Conclusion and Future Outlook

Legumes are now a core part of many plant‐based diets, but they also remain an important source of food allergens. This is largely because their major storage proteins are structurally robust, often resistant to digestion, and can cross‐react across different legume species. In this review, it was demonstrated that NTTs including high‐pressure processing, pulsed electric fields, cold plasma, and ultrasound offer a promising approach to reduce legume allergenicity by altering the protein structure in more targeted ways than conventional heating. Across studies, these treatments can reduce IgE reactivity by promoting unfolding, aggregation, fragmentation, or chemical modification of proteins, which can limit the accessibility of IgE‐binding epitopes. Across studies reviewed, IgE‐binding reductions range from 30% to 75% for single NTTs and 70% to 100% for combined approaches. Importantly, many reports also suggest that these approaches can preserve sensory quality and nutritional value better than intense thermal processing.

At the same time, the evidence makes it clear that results are not consistent across all legumes or all processing setups. Nevertheless, the variability observed depends on the legume species, protein matrices, and processing conditions; some allergenic epitopes appear to remain resistant even after processing. Where enzymes are used, effectiveness also depends on protease specificity and the presence of antinutritional factors, protease‐resistant components, or protease inhibitors (such as lectins), which may help explain why hydrolysis outcomes differ between legumes like chickpea, pea, and white bean.

Looking ahead, progress in this area will depend on moving from “proof of concept” to approaches that are more standardized and easier to translate into real food systems. This includes clearer reporting and harmonization of processing parameters, broader testing in realistic food matrices, and digestion models that better reflect human physiology. Clinical validation remains especially important because reductions in IgE binding do not always translate directly into reduced clinical risk. Future research should focus on a stepwise validation pipeline beginning with standardized in vitro assays, followed by ex vivo immune‐cell activation studies, animal models where appropriate, and ultimately randomized clinical trials or supervised oral food challenges to establish whether reductions in IgE binding translate into clinically meaningful reductions in allergic reductions. Finally, combining two or more NTTs, especially with enzymatic hydrolysis, appears to be one of the most practical paths forward, and future work should focus on designing legume‐specific protocols based on protein structure, matrix effects, and processing goals. With stronger mechanistic understanding and better validation, NTT‐based strategies could become a reliable route toward safer, more widely acceptable legume‐based foods.

## Author Contributions

**Viateur Ngabonzima:** investigation, writing – original draft, methodology, software, formal analysis, data curation, resources, writing – review and editing. **Flora G. C. Ekezie:** conceptualization, writing – review and editing. **Alberta N. A. Aryee:** writing – review and editing. **John O. Onuh:** conceptualization, investigation, funding acquisition, writing – original draft, writing – review and editing, visualization, validation, methodology, project administration, supervision, resources, formal analysis, software.

## Funding

The funding support for this study was provided by the George Washington Carver Agricultural Experiment Station, Tuskegee University through the USDA/NIFA Evan Allen grant.

## Conflicts of Interest

The authors declare no conflicts of interest.

## Data Availability

The data that support the findings of this study are available from the corresponding author upon reasonable request.
